# Responses of ‘Flordaguard’ and ‘MP‐29’ *Prunus* spp. rootstocks to hypoxia and high root zone temperature

**DOI:** 10.1002/pei3.70007

**Published:** 2024-10-14

**Authors:** Logan Richmond‐Cosie, Bruce Schaffer, Muhammad A. Shahid, José X. Chaparro, Ali Sarkhosh

**Affiliations:** ^1^ Horticultural Sciences Department University of Florida Gainesville Florida USA; ^2^ Tropical Research and Education Center University of Florida Homestead Florida USA; ^3^ North Florida Research and Education Center University of Florida Quincy Florida USA

**Keywords:** oxygen depletion, severe weather, soil environment effects, thermal tolerance, tree fruit resilience

## Abstract

Selecting the right rootstock is crucial for successful fruit production and managing both biotic and abiotic stresses in commercial fruit orchards. To enhance the resilience of peach orchards, this study evaluated the physiological and biochemical responses of *Prunus* spp. rootstocks , ‘Flordaguard’ and ‘MP‐29,’ under normoxia (sufficient oxygen content) or short‐term hypoxia (low‐oxygen content) and ambient or high temperature (40°C) in the root zone. Physiological responses measured were net photosynthesis, stomatal conductance, transpiration, intercellular CO_2_ concentration, water use efficiency, the leaf chlorophyll index, and the maximum potential quantum efficiency of photosystem II. The leaf concentrations of nitrogen, phosphorus, potassium, magnesium, calcium, sulfur, boron, zinc, manganese, iron, and copper were also analyzed. Reactive oxygen species (ROS) and antioxidants analyzed were superoxide dismutase (SOD) activity, peroxidase (POD) activity, catalase (CAT) activity, ascorbate peroxidase (APX) activity, glutathione peroxidase (GPX) activity, proline content, glycine betaine content (GB), lipid peroxidation (LPO), superoxide (O_2_^−^) concentration, and hydrogen peroxide (H_2_O_2_) concentration. When subjected to root zone hypoxia or high temperature individually, ‘MP‐29’ performed better physiologically than ‘Flordaguard’. However, when root zone hypoxia and high temperature were combined, ‘MP‐29’ performed better biochemically with enhanced antioxidant activity, osmolyte content, and nutrient absorption. Nutrient analysis of leaves revealed that ‘MP‐29’ had higher N, P, K, Ca, and B concentrations than ‘Flordaguard’. Consequently, ‘MP‐29’ demonstrated greater tolerance to short‐term exposure to the combined effects of high root zone temperature and hypoxia. This research contributes to identifying a suitable rootstock within the *Prunus* genus able to withstand root zone conditions that often result from severe weather events commonly experienced in Florida and other parts of the world.

## 1 INTRODUCTION

Exposure to adverse environmental and edaphic conditions such as hypoxia (low soil oxygen) or temperatures deviating from acceptable root zone temperature thresholds can trigger a systemic stress response resulting in reduced productivity and eventual mortality in plants (Bhusal et al., 2020; Teskey et al., 2014; Wu et al., 2022). Plant species and rootstocks/cultivars have been found to drive variations in tolerance to these stresses through differences in photosynthetic activity, assimilate partitioning, and antioxidant enzyme activity (Bhusal et al., 2020; Pimentel et al., 2014; Ranney, 1994; Teskey et al., 2014; Wu et al., 2022). The intensification of environmental conditions, such as rising temperatures and severe precipitation events, effectuated by climate change, has led to research on crop biochemical stress responses and the impact on physiological responses which may facilitate tolerance to root zone hypoxia and heat stress. These biochemical responses include increased antioxidant activity, osmolyte accumulation, and chaperone production as result of high temperatures (30–35°C) (Masouleh & Sassine, 2020). Such responses manage oxidative stress and protect proteins within plant cells, reducing the risk of cellular death due to environmental stressors.

Heavy precipitation can result in water saturation of available pore space in the soil, leading to a decrease in root gas exchange (Dat et al., 2006). This reduction in gas exchange decreases root permeability and limits nutrient and water uptake (Kreuwieser & Rennenberg, 2014). During periods of high air temperatures, plants may upregulate transpiration to maintain internal plant temperatures, which can further exacerbate water stress if soil becomes waterlogged. This scenario can hamper photosynthetic activity, decreasing available cellular energy stores (Araki et al., 2012; Rogers, 1993). In response to oxygen and water deficit, roots of *Prunus* spp. often elongate to reach available oxygen in the soil. However, this growth comes at a cost to already depleted carbohydrates due to the shallow‐rooted nature of *Prunus* rootstocks (Rogers, 1993). Nutritional requirements to mitigate stress may present obstacles to maintaining homeostasis, sustain photosynthesis, and support root growth and development. Without effective gas exchange within the plant, mitochondrial respiration is inhibited, leading to an upregulation of anaerobic respiration, which decreases ATP synthesis (Dat et al., 2006; Parent et al., 2008). The increase in fermentative pathways leads to the production of a surplus of fermentation by‐products, such as lactic acid, acetaldehyde, and/or reactive oxygen species (ROS) (Amador et al., 2009; Fukao & Bailey‐Serres, 2004), resulting in cellular acidosis and eventual necrosis (Parent et al., 2008). Symptoms similar to drought including wilting, chlorosis, and defoliation are observed when plant cells are under hypoxia stress (Kunert et al., 2021; Smit et al., 1989). The reduction in cellular turgor causes the wilting of plant tissue, while discoloration and loss of leaves limits photosynthetic activity, which relies on light absorption by chlorophyll to maintain ATP production (Meyer, 1998).

Oxidative stress jeopardizes homeostasis and plant growth and development. Oxidative stress refers to the downregulation of antioxidants such as ascorbate peroxidase (APX), catalase (CAT), glutathione peroxidase (GPX), peroxidase (POD), and superoxide dismutase (SOD) that work to scavenge harmful ROS (Alscher, 2002; Caverzan et al., 2012; Chaki et al., 2020). If there is an imbalance in ROS sequestration and reduction, these compounds will initiate lipid peroxidation, enzyme denaturation, cell death, and premature leaf senescence (Hasanuzzaman et al., 2013; Jackson & Colmer, 2005; Masouleh & Sassine, 2020). Enzymatic antioxidants such as APX, CAT, GPX, POD, and SOD, convert harmful ROS, such as hydrogen peroxide (H_2_O_2_), into non‐toxic water and oxygen gas (Hasanuzzaman et al., 2013; Masouleh & Sassine, 2020). GPX is also important for the remediation of lipid peroxidation, as it also reduces lipid peroxides into lipid alcohols to preserve structure and function of cellular membranes (Halliwell, 1987). The increased activity of enzymatic antioxidants is considered a potential stress tolerance response, as antioxidants play a vital role in maintaining cellular oxidative balance.

Osmolytes, such as proline and glycine betaine (GB), function as another line of defense against oxidative stress in plant cells (Sharma et al., 2019). These organic compounds protect cells from oxidative stress by preserving cell turgidity, unfolding damaged proteins, and stabilizing thylakoid membranes (Jaleel et al., 2007; Patade et al., 2013). These osmolytes maintain osmotic potential by conducting osmoregulation and by controlling the water content of the cells. Moreover, they help to stabilize macromolecules, maintain protein structures, and increase protein folding efficiency (Sharma et al., 2019). In addition to osmolytes, antioxidants play a crucial role in mitigating oxidative stress in plant cells. Antioxidants scavenge ROS, thereby reducing ROS damaging effects (Sharma et al., 2019). Some of the important antioxidants in plants include ascorbate, glutathione, tocopherols, and carotenoids (Choudhury et al., 2016). Ascorbate and glutathione help to maintain the redox state of the cells, while tocopherols and carotenoids help to quench ROS (Sharma et al., 2019).

Studies of the thermotolerance of *Prunus* spp. have shown variation in responses within rootstocks/cultivars (Bonomelli et al., 2009; Bonomelli et al., 2012; Hao et al., 2011; Li et al., 2020). However, those studies have yet to include root zone temperatures that reflect those experienced in Florida during the hot summer months. Florida summer months are of particular interest in this study, as higher air temperatures are experienced during Florida rain and hurricane seasons. Florida's hurricane season, spanning from June to November, coincides with the summer months marked by extended daylight and intense sunlight, resulting in increased atmospheric temperatures. Thus, in the summer months, after heavy rainfall, roots of *Prunus* spp. are often exposed to a short‐term (2–4 days) period of combined heat and hypoxia stresses. Mismanagement of irrigation and groundcover can lead to soil temperatures exceeding surrounding air temperatures, primarily with the top 20–30 cm of soil depth (Duncan et al., 1992; Zhan et al., 2019). Extended periods of high air temperatures may increase soil temperatures, adversely affecting crop growth and nutrient absorption (Onwuka, 2016). Due to the shallow rooted nature of peach rootstocks, peach trees are particularly vulnerable to the detrimental effects of high ambient temperatures (Reighard et al., 2001; Xiao et al., 2020). However, the combined effects of high root zone temperatures and hypoxia on physiology and growth of *Prunus* spp. rootstocks has not been reported. Heat stress during flooding and post‐flooding recovery periods can further compound root and tree damage.

For Florida's low‐chill peach production, the University of Florida (UF), Institute of Food and Agricultural Sciences through research conducted by the UF Horticultural Science Stone Fruit Extension Laboratory currently recommends *Prunus* rootstock ‘Flordaguard’ due to its tolerance to damaging root‐knot nematode species, including *Meloidogyne floridensis*, *M. javanica*, and *M. incognita*. Root‐knot nematodes, particularly *M. floridensis*, impair peach trees by inducing root galls, compromising vascular function, reducing tree vigor, and decreasing fruit quantity and quality (Maquilan et al., 2018). The *Prunus* rootstock ‘MP‐29,’ also exhibits resistance to these nematodes, and *Armillaria* root rot caused by the fungus *Armillaria* spp., that attacks both living and dead root tissues and can impact above‐ground tree architecture causing trees to lean and eventually tree collapse (Kim et al., 2022; IFAS Communications, 2023). ‘MP‐29’ is also resistant to peach tree short life, which is defined by the abrupt terminal collapse of young peach trees, resulting from the interaction of several factors including nutrient uptake, bacterial and fungal infection, and cultural practices (Beckman & Nyczepir, 2004).

The two rootstocks were chosen for this study due to ‘Flordaguards production recommendation status, while ‘MP‐29′ is a more recently developed rootstock that has shown promise in the field. There is interest in determining which rootstock may be better suited to tolerate severe weather events, which are inevitable in Florida due its vulnerable peninsular shape surrounded by the warm waters of the Gulf of Mexico and Atlantic Ocean.

While ‘MP‐29’ appears to be more tolerant to root zone hypoxia than ‘Flordaguard’ under ambient root zone temperatures in Florida, the relative tolerance of these rootstocks to high root zone temperature or a combination of high root zone temperature and hypoxia has not been investigated. To ensure sustainability of orchard operations, it is crucial to develop and effectively implement genotypes that are suitable to specific production environments. The objectives of this study were to compare leaf physiological and biochemical responses of *Prunus* spp. rootstocks, ‘Flordaguard’ and ‘MP‐29’, to short‐term exposure to and recovery from hypoxia and high temperature (40 °C) in the root zone. The hypothesis evaluated was that ‘Flordaguard’ and ‘MP‐29’ differ in their biochemical and physiological responses to and recovery from hypoxia and high temperature in the root zone.

## 2 MATERIALS AND METHODS

### 2.1 Plant material and experimental conditions

The study was conducted in a greenhouse at the University of Florida in Gainesville, FL, USA. Eighty plants, propagated by tissue culture, 40 of each rootstock, ‘Flordaguard’ and ‘MP‐29,’ were obtained from commercial propagation company, Agristarts Inc. (Apopka, FL, USA), and maintained in a closed, deep‐water culture hydroponic system. The hydroponic system consisted of 13.2‐liter buckets holding four plants each, two ‘Flordaguard’ and two ‘MP‐29’. Plants were maintained in the system for 8 weeks to allow for acclimation to the greenhouse environment and nutrient solution. The hydroponic nutrient solution was formulated according to Hoagland and Arnon (1950) and the pH was adjusted to 6.5. Once plants reached 33 cm in height with the presence of new growth denoting successful acclimation to the greenhouse environment and hydroponic system, treatments were initiated.

The ambient temperature, relative humidity, and dew point in the greenhouse were monitored before and during the experiment with HOBO*®* U23 Pro v2 temperature/relative humidity dataloggers (Onset Computers, Bourne, MA, USA). The ambient air temperature in the greenhouse ranged from 18.7 to 39.2°C with a daily mean of 28.9°C. Relative humidity ranged from 33.3 to 96.7% with a daily mean of 65.0%. Dew point ranged from 16.5 to 25.6°C with a daily mean of 21.5°C. A HOBO® Water Temperature Pro v2 datalogger (Onset Computers, Bourne, MA, USA) was placed in a randomly selected bucket in each treatment to monitor water temperature during the experiment.

The experimental design was a factorial design, consisting of two rootstocks (‘Flordaguard’ and ‘MP‐29’, *Prunus persica* and *P. umbellate* × *P*. *persica*, respectively) and four root zone conditions (treatments): (1) normoxia (sufficient oxygen in the root zone) with ambient root zone temperatures, (2) hypoxia (low oxygen in the root zone) and ambient root zone temperature, (3) high soil temperature (40°C) and normoxia, and (4) hypoxia and high root zone temperature. There were five replicates of each treatment per rootstock arranged in a complete block design with each treatment of each rootstock in each of the five blocks. The five blocks accounted for the differing amount of sunlight along the two greenhouse benches used. Treatments were maintained for 4 days. After 4 days, all plants were returned to control conditions (normoxia with ambient root zone temperature). The recovery of each rootstock after treatments ceased was determined by measuring physiological variables from days five through eight.

For the ambient root zone temperature treatments, root zone temperatures were acclimated to the greenhouse environment and not altered. Normoxic root zone conditions (6–8 O_2_ mg/H_2_O L) were maintained by aerating the nutrient solution with oxygen gas (O_2_) through an air compressor outfitted with three air stones per bucket. Hypoxic root zone conditions (≤3 O_2_ mg/H_2_O L) were maintained by pumping 100% ultra‐high purity nitrogen gas (N_2_) into the nutrient solution for 8 h per day following the methods outlined by Gil et al. (2009) and Butler and Rickard (1994). Plants were exposed to high temperature (40°C) in the root zone using a model 200 W submersible titanium water heater (Hygger, Renton, WA, USA).

**FIGURE 1:**
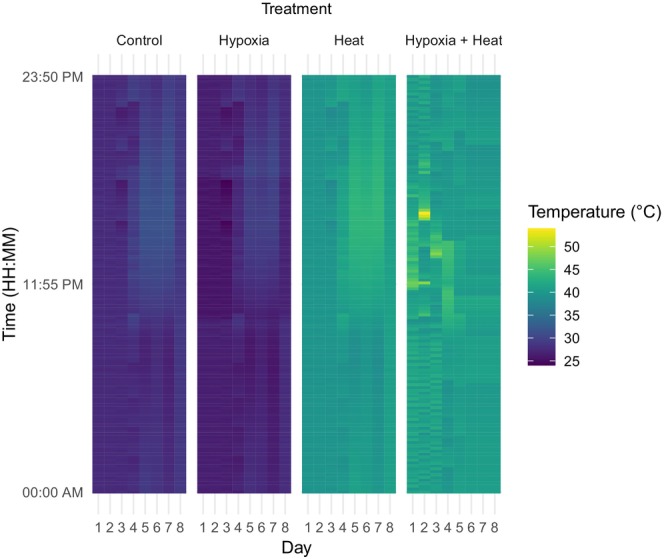
Mean daily temperature (°C) of the nutrient solution.

**FIGURE 2:**
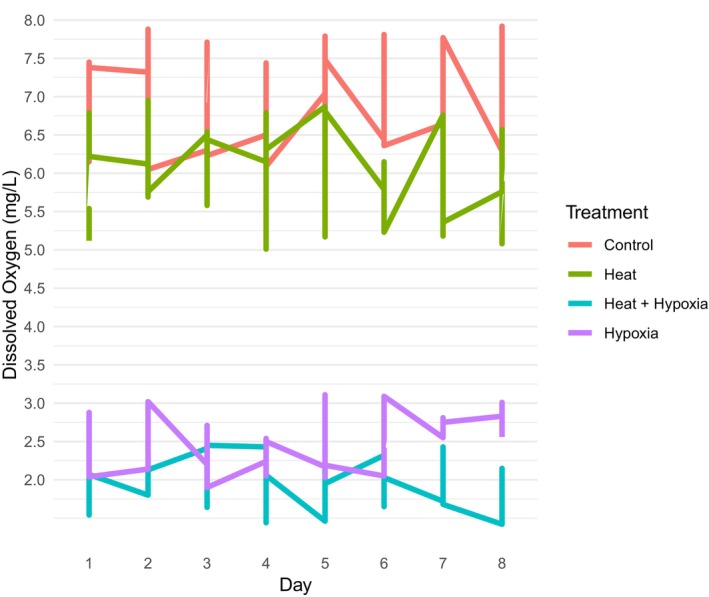
Mean daily dissolved oxygen content (mg/L) of the nutrient solution.

The pH and dissolved oxygen content in the hydroponic solution were measured before and during the experiment with a multiparameter water meter (model Plus 1700/1725, YSI Inc., Yellow Springs, OH, USA). Temperatures of the nutrient solution for non‐heated treatments ranged from 26.0 to 31.8°C with a daily mean of 28.9°C (Figure 1). Temperatures of the nutrient solution for the heated treatment ranged from 38.0 to 43.8°C with a daily mean of 40.9°C (Figure 1). Temperatures of the nutrient solution for the hypoxia plus heat treatment ranged from 39.0 to 53.9°C with a daily mean of 46.4°C (Figure 1). The pH of the nutrient solution for all treatments ranged from 6.4 to 6.5 with a daily mean of 6.4. Dissolved root zone oxygen content in the control (normoxia, non‐heated) treatment ranged from 6.05 to 7.9 mg/L with a daily mean of 6.9 mg/L (Figure 2). Dissolved root zone oxygen content in the hypoxia, non‐heated treatment ranged from 1.9 to 3.1 mg/L with a daily mean of 2.5 mg/L (Figure 2). Dissolved root zone oxygen content in the normoxia, heated treatment ranged from 5.0 to 7.0 mg/L with a daily mean of 6.0 mg/L (Figure 2). Dissolved oxygen content in the hypoxia, heated treatment ranged from 1.4 to 2.4 mg/L with a daily mean of 2.0 mg/L (Figure 2).

### 2.2 Physiological measurements

Leaf‐level physiological processes (net photosynthesis, stomatal conductance, transpiration, and intercellular CO_2_ concentration) were measured in two mature (fully expanded), undamaged leaves per plant with an infrared leaf gas analyzer (model LI‐6800XT, LiCor Biosciences, Lincoln, NE, USA). The aforementioned physiological processes were measured daily between 10:00 and 12:00 HR at a photosynthetic photon flux density in the leaf cuvette of 1200 μmol m^–2^ s^–1^, an air flow rate into the leaf cuvette of 400 μmol s^–1^, and a reference CO_2_ concentration of 400 μmol CO_2_ mol^–1^. Water use efficiency was calculated as net photosynthesis divided by transpiration.

The maximum potential quantum efficiency of photosystem II was measured as the ratio of variable to maximum chlorophyll fluorescence (Fv/Fm) in two mature (fully expanded), undamaged leaves per plant with an OS30P chlorophyll fluorometer (Opti‐Sciences Inc., Hudson, NH, USA), after dark adaptation for 20 min. The leaf chlorophyll index (LCI) was measured with a SPAD 502DL plus chlorophyll meter (Konica Minolta Sensing Americas Inc., Ramsey, NJ, USA).

### 2.3 ROS and antioxidant analyses

After the recovery period (day 8), 1 g of fully expanded, mature leaves was harvested from both plants of each rootstock in each block of each treatment (40 replicates), totaling 40 leaf samples. Leaves were harvested using sterile pruners, placed in individual plastic bags and stored at −80°C for 3 days before being sent to the University of New Hampshire for analyses. Metabolites and enzymes analyzed included SOD, POD, CAT, APX, GPX, proline content, GB, LPO, O_2_^−^, H_2_O_2_ concentrations. Methods for ROS and antioxidant extraction and determination were described in detail by McGee et al. (2021).

For antioxidant analysis, 0.5 g of frozen leaf tissue and 5 mL of 50 mM chilled phosphate buffer (pH 7.8) were homogenized, then centrifuged at 15,000*g* for 20 min at 4°C. The supernatant was processed for enzymatic activity quantification. SOD activity was evaluated following Giannopolitis and Ries (1977) by calculating the inhibition of nitro blue tetrazolium (NBT) photoreduction. CAT and POD activities were evaluated following Maehly and Chance (1954) with modifications: (1) the CAT assay used a 3 mL solution of 50 mM phosphate buffer, 5.9 mM H_2_O_2_, and 0.1 mL of enzyme extract, recording absorbance at 240 nm recorded every 20 s; (2) the POD assay used a 3 mL solution of 50 mM phosphate buffer, 20 mM guaiacol, 40 mM H_2_O_2_, and 0.1 mL of enzyme extract, recording absorbance at 470 nm recorded every 20s. APX activity was evaluated following Nakano and Asada (1981) with a 1 mL reaction solution of 50 mM potassium phosphate buffer, 0.5 mM ascorbate, 0.4 mM H_2_O_2,_ and 50 μL of enzyme extract, with nmol of ascorbate decomposed per gram of protein at 290 nm for 30°C marking APX activity. GPX activity was assessed according to Urbanek et al. (1991) where the reaction solution containing 25 μL of enzyme extract, 50 mM potassium phosphate buffer, 20 guaiacol, and 20 mM H_2_O_2_, was incubated for 30°C for 12 min.

O_2_^−^ was evaluated following Elstner and Heupel (1976) with a mixture of 0.5 g of the frozen leaf tissue, 0.5 mL of phosphate buffer, 1 mL of xanthine oxidase, and 0.1 mL of hydroxyl ammonium chloride, incubated at 25°C for 20 min. Then, a 0.5 mL aliquot of the mixture was combined with 0.5 mL sulfanilic acid and 0.5 mL of α‐nepthylamine, kept at room temperature for about 20 min, and assessed for optical density at 530 nm. H_2_O_2_ was assessed according to Patterson et al. (1984), wherein 1 g of the leaf tissue was mixed with 2 mL acetone, treated with a titanium reagent and 17 M of ammonia solution, precipitated, separated, washed in acetone, and dissolved in H_2_SO_4_. Finally, absorbance of the solution was evaluated at 410 nm.LPO was measured following Heath and Packer (1968) by mixing equal volumes of enzyme extract and 0.5% (w/v) TBA solution containing 20% (w/v) tri‐chloroacetic acid (TCA) and heating this mixture at 95°C for 30 min followed by rapid cooling in an ice bath. This mixture was centrifuged at 3000*g* for 10 min, with the supernatant measured for absorption at 532 and 600 nm. The proline and GB content were measured following the methodology described by Bates et al. (1973) and Grieve and Grattan (1983), respectively.

### 2.4 Nutrient analyses

Immediately after the recovery period (day 8), 5 g of fully expanded, mature leaves were harvested from both plants in each block of each treatment, for a total of 40 leaf samples. Leaves were harvested using sterile pruners and stored in individual plastic bags. Samples were placed in a desiccation oven at 55ºC for 48 h before being sent to Waters Agricultural Laboratories Inc. (Camilla, GA, USA) for nutrient analyses. The concentrations of nitrogen (N), phosphorous (P), potassium (K), magnesium (Mg), calcium (Ca), sulfur (S), boron (B), zinc (Zn), manganese (Mn), iron (Fe), and copper (Cu) were determined following the procedure outlined by Shahkoomahally et al. (2020) and conducted by Waters Agricultural Laboratory, based on the methods in Isaac and Johnson (1985). Dried leaf samples were ground using a Wiley Model 4 Mill (Thomas Scientific, Swedesboro, NJ, USA) until able to filter through a 1.00 mm mesh size. From the ground sample material, 0.5 g of leaf tissue was added to a tube, followed by the addition of 5 mL of 70% nitric acid. The tubes were heated at 90°C for 90 min. Then, 4 mL of 30% hydrogen peroxide was added, and following the cessation of the reaction, the samples were heated again at 95 °°C for 20 min, then cooled for 2 min. The tubes were then diluted to 50 mL with deionized water, mixed, filtered, and transferred to ICP tubes for analysis. The tissue analysis was performed using an ICAP‐Open Vessel Wet Digestion Digi Block 3000.

### 2.5 Statistical analyses

The factorial design allowed for the investigation of both the main effects of each factor (rootstock and root zone condition) and their interaction effects on the studied variables. By using a factorial design, the study could comprehensively assess how different combinations of rootstock and rootzone conditions influence the physiological and biochemical measurements assessed. All dependent variables were analyzed by a two‐way analysis of variance (ANOVA) to assess statistical interactions between treatments and rootstocks. A one‐way ANOVA and a Waller–Duncan *K*‐ratio test were used to compare treatment means while controlling for the comparison‐wise‐error‐rate. An un‐paired *t*‐test was used to compare the mean values between rootstocks. All analyses were done using SAS statistical analysis software (version 9.4, SAS Institute Inc., Cary, NC, USA).

## 3 RESULTS

### 3.1 Leaf gas exchange

**FIGURE 3:**
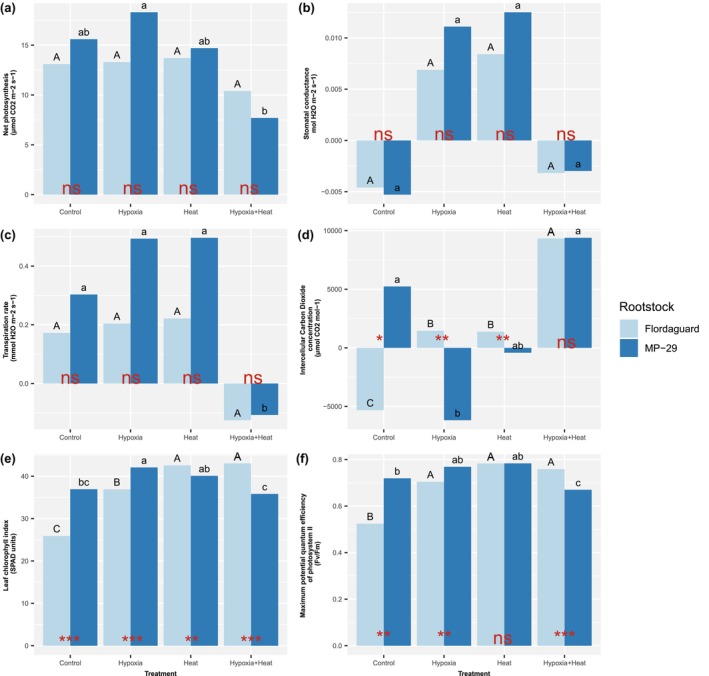
Effect of rootstock and rootzone hypoxia and heat treatment on physiological variables in the leaves of *Prunus* spp. rootstocks, ‘Flordaguard’ and ‘MP‐29’. The bold uppercase letters in the upper left‐hand corner of the figures indicate the different variables: (a) net photosynthesis, (b) stomatal conductance, (c) transpiration, (d) intercellular CO_2_ concentration, (e) leaf chlorophyll index, (f) maximum quantum efficiency of photosystem II. Different letters indicate statistically significant differences among treatments by rootstock according to a Duncan–Waller *K*‐ratio test (*p* ≤ .05). Upper‐case letters denote ‘Flordaguard’ and lower‐case letters for ‘MP‐29’. Means with ‘*,’ ‘**,’ ‘***’ indicate statistically significant differences at *p* ≤ .05, .01, or .001, respectively; and ns indicates no significant difference according to a non‐paired *t*‐test.

Normoxic plants exhibited significantly higher net photosynthesis compared to hypoxic plants for both ‘Flordaguard’ and ‘MP‐29’ rootstocks in the heated and non‐heated treatments (Figure 3). In the normoxic, non‐heated treatment, ‘MP‐29’ had significantly higher net photosynthesis than ‘Flordaguard’. In the hypoxia, high temperature treatment, ‘MP‐29’ had significantly higher net photosynthesis than ‘Flordaguard’. However, there was no statistically significant difference in net photosynthesis between ‘Flordaguard’ and ‘MP‐29’ in the hypoxic, high temperature treatment (Figure 3).

Stomatal conductance was significantly higher in normoxic plants compared to hypoxic plants for both rootstocks in both non‐heated and heated treatments (Figure 3). However, there was no statistically significant difference in stomatal conductance between ‘Flordaguard’ and ‘MP‐29’ in the normoxic, non‐heated treatment, the, hypoxic non‐heated treatment, or the hypoxic, heated treatment (Figure 3).

In the heated treatments, transpiration of ‘Flordaguard’ and ‘MP‐29’ was significantly higher in normoxic than hypoxic plants (Figure 3). In contrast, within the non‐heated treatments, transpiration was significantly higher in hypoxic than normoxic plants for both rootstocks (Figure 3). In the normoxic, non‐heated treatment, there was no statistically significant difference in transpiration between ‘Flordaguard’ and ‘MP‐29’ (Figure 3). However, in the hypoxic, heated treatment, transpiration was significantly lower in ‘Flordaguard’ than ‘MP‐29’ (Figure 3). No significant difference in transpiration was observed between ‘Flordaguard’ and ‘MP‐29’ in the normoxic, heated treatment or the hypoxia, heated treatment (Figure 3).

In both heated treatments, intercellular CO_2_ concentration of ‘Flordaguard’ and ‘MP‐29’ was significantly higher in hypoxic than normoxic plants (Figure 3). Within the non‐heated treatments, ‘Flordaguard’ had a higher intercellular CO_2_ concentration in hypoxic than normoxic plants, whereas ‘MP‐29’ had a higher concentration in hypoxic plants (Figure 3). In the normoxic, non‐heated treatment, the intercellular CO_2_ concentration was significantly higher in ‘Flordaguard’ than in ‘MP‐29’ (Figure 3). However, in the hypoxic, heated treatment, no significant difference was observed in intercellular CO_2_ concentration between rootstocks (Figure 3). There were no significant differences in water use efficiency between hypoxic and normoxic plants within the heated or non‐heated treatments for ‘Flordaguard’ or ‘MP‐29’.

### 3.2 Leaf chlorophyll index and the maximum potential quantum efficiency of photosystem II (Fv/Fm)

In the heated treatments, the maximum potential quantum efficiency of photosystem II (Fv/Fm) in both ‘Flordaguard’ and ‘MP‐29’ was significantly higher in normoxic than hypoxic plants (Figure 3). Similar results were observed in the non‐heated treatments, where Fv/Fm was significantly higher in normoxic than the hypoxic plants for each rootstock (Figure 3). In the normoxic, non‐heated treatment, Fv/Fm was significantly lower in ‘Flordaguard’ than in ‘MP‐29’ (Figure 3). In the hypoxic, non‐heated treatment, Fv/Fm was lower in ‘Flordaguard’ compared to ‘MP‐29’ (Figure 3). However, in the normoxic, heated treatment, no significant difference in Fv/Fm was observed between rootstocks (Figure 3). In the hypoxic, heated treatment, Fv/Fm was significantly higher in ‘Flordaguard’ than ‘MP‐29’ (Figure 3).

For ‘Flordaguard,’ there were no significant differences in the leaf chlorophyll index between hypoxic and normoxic plants in the non‐heat treatments (Figure 3). However, within the heated treatments, the leaf chlorophyll index of ‘MP‐29’ was significantly lower in hypoxic than normoxic plants, suggesting a potential sensitivity of ‘MP‐29’ to hypoxic conditions (Figure 3). In contrast, within the non‐heated treatments for both rootstocks, the leaf chlorophyll index was significantly higher in hypoxic than normoxic plants (Figure 3). ‘Flordaguard had a significantly lower leaf chlorophyll index than ‘MP‐29′ (Figure 3). However, in the normoxic, heated treatment and both hypoxic treatments, the leaf chlorophyll index was significantly lower in ‘MP‐29′ than in ‘Flordaguard’ (Figure 3).

### 3.3 Leaf nutrient concentrations

**FIGURE 4:**
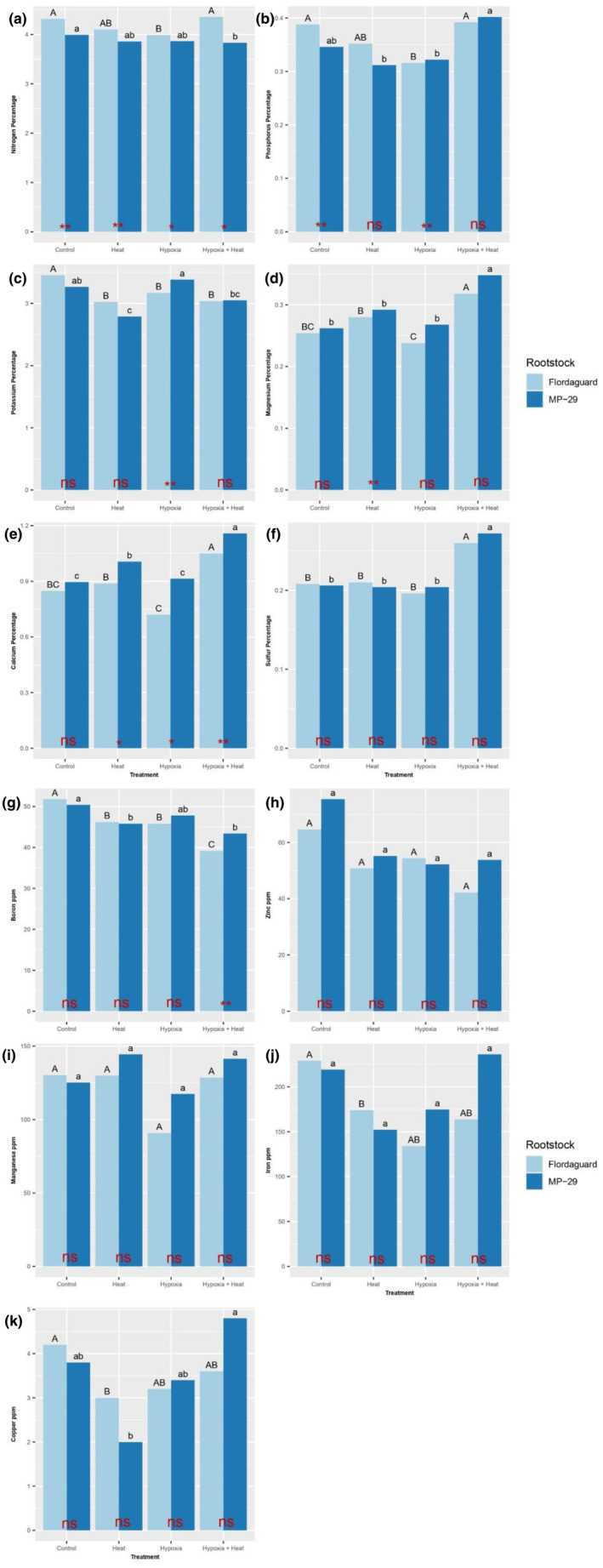
Effect of rootstock and root zone hypoxia and heat treatment on leaf nutrient concentrations of *Prunus* spp. rootstocks, ‘Flordaguard’ and ‘MP‐29’. (a) nitrogen percentage, (b) phosphorous percentage, (c) potassium percentage, (d) magnesium percentage, (e) calcium percentage, (f) sulfur percentage, (g) boron ppm, (h) zinc ppm, (i) manganese ppm, (j) iron ppm, and (k) copper ppm. For detailed explanations of graph elements and statistical annotations, please see Figure 3 caption.

In both heated treatments, the N concentration in Flordaguard was significantly higher in hypoxic plants than normoxic plants (Figure 4). Conversely, within both heated treatments, there was no significant difference in N concentration in ‘MP‐29’ between hypoxic and normoxic plants (Figure 4). For ‘Flordaguard,’ in both non‐heated treatments, the N concentration was significantly higher in normoxic than hypoxic plants (Figure 4). For ‘MP‐29,’ there were no significant differences in the N concentration between hypoxic and normoxic plants (Figure 4). For all treatments, ‘Flordaguard’ consistently exhibited higher N concentrations than ‘MP‐29’ (Figure 4).

In the heated treatments for ‘Flordaguard,’ the P concentration was significantly higher in hypoxic than normoxic plants, whereas there was no significant difference in ‘MP‐29’ rootstocks (Figure 4). In non‐heated treatments, the P concentration was significantly higher in normoxic than hypoxic plants of ‘Flordaguard,’ whereas there was no significant difference in P concentrations between hypoxic and normoxic plants of ‘MP‐29’ (Figure 4). In the normoxia, non‐heated treatment, and the hypoxia treatment, the P concentration was significantly higher in ‘Flordaguard’ compared to ‘MP‐29’ (Figure 4). However, in the hypoxia, non‐heated treatment and the hypoxia, heated treatments, there was no significant difference in P concentration between ‘Flordaguard’ and ‘MP‐29’ (Figure 4).

For ‘Flordaguard,’ in the heated treatments, there was no significant difference in the K concentration between hypoxic and normoxic plants, whereas in ‘MP‐29,’ the K concentration was significantly higher in hypoxic than normoxic plants (Figure 4). In both non‐heated treatments, the K concentration was significantly higher in normoxic than that in hypoxic ‘Flordaguard plants, whereas for ‘MP‐29,’ there was no significant difference in the K concentration between hypoxic and normoxic plants (Figure 4). For the normoxia treatment, the hypoxia, non‐heated treatment, or the hypoxia, heated treatment, there was no significant difference in the K concentration between the two rootstocks (Figure 4). However, in the hypoxia, heated treatment, the K concentration was significantly higher in ‘Flordaguard’ than that in ‘MP‐29′ (Figure 4).

In the heated treatments for both rootstocks, the Mg concentration was significantly higher in hypoxic than that in normoxic plants (Figure 4). In both non‐heated treatments, the Mg concentration was significantly higher in normoxic than that in hypoxic ‘Flordaguard’ plants, whereas the Mg concentration was significantly higher in hypoxic than that in normoxic ‘MP‐29’ plants (Figure 4). In the normoxia, non‐heat treatment, the normoxia, heated treatment, or the hypoxia, heated treatment, there was no significant difference in the Mg concentration between the two rootstocks (Figure 4). However, in the hypoxia, non‐heated treatment, the Mg concentration was significantly higher in ‘MP‐29’ compared to ‘Flordaguard’ (Figure 4).

In both heated treatments for both rootstocks, the Ca concentration was significantly higher in hypoxic than normoxic plants (Figure 4). In both non‐heated treatments, for ‘Flordaguard’, the Ca concentration was significantly higher in normoxic than hypoxic plants (Figure 4). In contrast, within the non‐heated treatments for ‘MP‐29,’ there was no significant difference in the Ca concentration between hypoxic and normoxic plants (Figure 4). In the normoxia, non‐heated, treatment, there was no significant difference in Ca concentrations between the two rootstocks (Figure 4). However, in non‐heated, hypoxia treatment, the heated normoxia treatment, and the heated, heated treatment, the Ca concentration was significantly higher in ‘MP‐29’ than in ‘Flordaguard’ (Figure 4).

In both heated treatments for both rootstocks, the S concentration was significantly higher in hypoxic than normoxic plants (Figure 4). In both non‐heated treatments, the S concentration was significantly higher in normoxic than hypoxic ‘Flordaguard’ plants, whereas there was no significant difference in the S concentration between hypoxic and normoxic ‘MP‐29’ plants (Figure 4). In all treatments, there was no significant difference in the S concentration between ‘Flordaguard’ and ‘MP‐29’ (Figure 4).

In both heated treatments for both rootstocks, the B concentration was significantly higher in normoxic than hypoxic plants (Figure 4). In the non‐heated treatments for both rootstocks, the B concentration was significantly higher in normoxic than hypoxic plants (Figure 4). In the normoxia, non‐heated the hypoxia non‐heated treatment, and the normoxia, heated treatment, there was no significant difference in the B concentration between ‘Flordaguard’ and ‘MP‐29’ (Figure 4). However, in the hypoxia, heated treatment, the B concentration was significantly higher in ‘MP‐29’ compared to ‘Flordaguard’ (Figure 4).

In both the heated and non‐heated treatments, there was no significant difference in the Zn concentration between hypoxic and normoxic plants (Figure 4). Also, in all treatments, there was no significant difference in the Zn concentration between ‘Flordaguard’ and ‘MP‐29’ (Figure 4).

In both the heated and non‐heated treatments for both rootstocks there was no significant difference in Mn concentrations between hypoxic and normoxic plants (Figure 4). Furthermore, in all treatments, there was no significant difference in the Mn concentration between ‘Flordaguard’ and ‘MP‐29’ (Figure 4).

In both the heated and non‐heated treatments for both rootstocks there was no significant difference in the Fe concentration between hypoxic and normoxic plants (Figure 4). Additionally, in all treatments, there was no significant difference in the Fe concentration between ‘Flordaguard’ and ‘MP‐29’ (Figure 4).

In both the heated and non‐heated treatments for both rootstocks, the Cu concentration was significantly higher in hypoxic than normoxic plants (Figure 4). However, in all treatments, there was no significant difference in the Cu concentration between ‘Flordaguard’ and ‘MP‐29’ (Figure 4).

### 3.4 Leaf ROS

**FIGURE 5:**
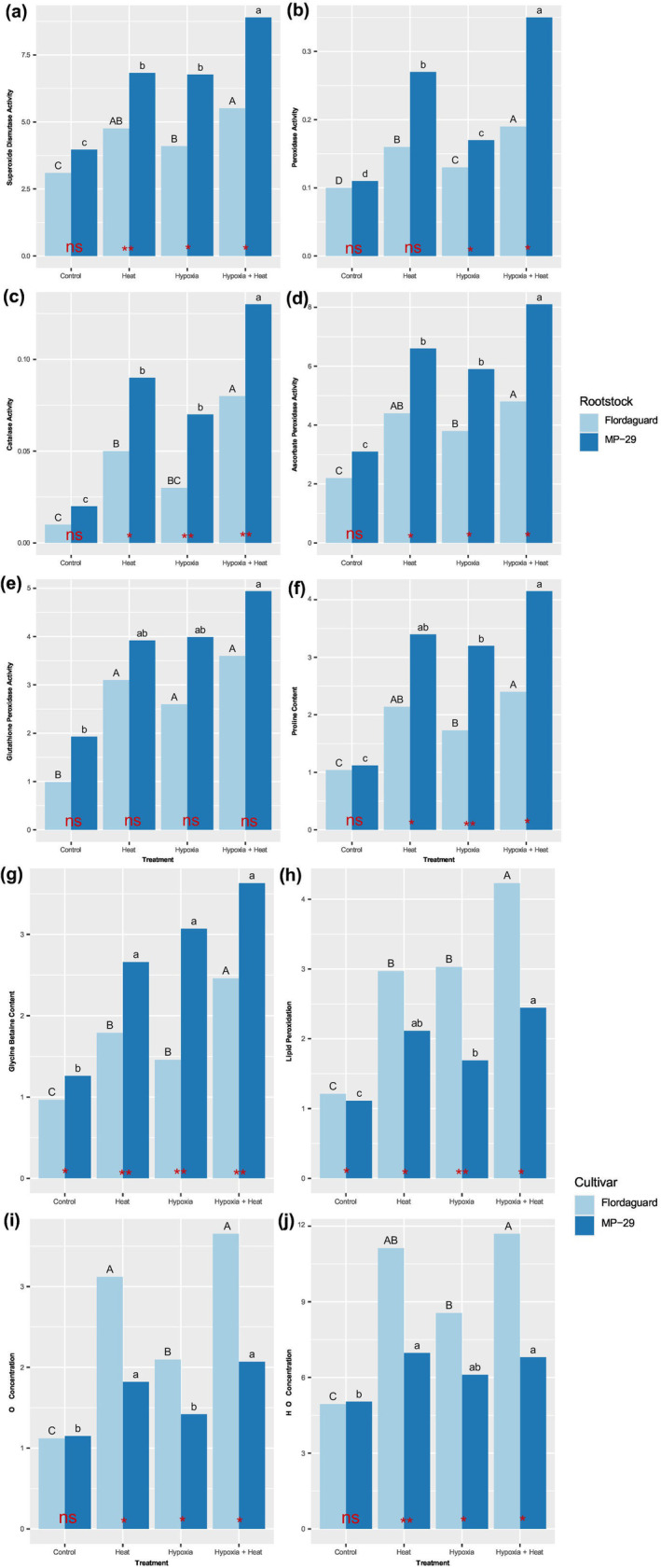
Effect of rootstock and root zone hypoxia and heat treatments on antioxidants in the leaves of *Prunus* spp. rootstocks, ‘Flordaguard’ and ‘MP‐29’. The bold uppercase letters in the left‐hand corner of the figures indicate the different parameter features. (a) superoxidase dismutase activity, (b) peroxidase activity, (c) catalase activity, (d) ascorbate peroxidase activity, (e) glutathione peroxidase activity, (f) proline activity, (g) glycine betaine accumulation, (h) lactoperoxidase activity, (i) O_2_ concentration, and (j) H_2_O_2_ concentration. For detailed explanations of graph elements and statistical annotations, please see Figure 3 caption.

In both the heated and non‐heated treatments for both rootstocks, O_2_^−^ levels were significantly higher in hypoxic than normoxic plants (Figure 5). In the hypoxia, heated treatment, there was a significant difference in O_2_^−^ levels between ‘Flordaguard’ and ‘MP‐29,’ with ‘Flordaguard’ having higher O_2_^−^ levels than ‘MP29’ (Figure 5).

In both heated treatments in ‘Flordaguard’, H_2_O_2_ levels were significantly higher in hypoxic than normoxic plants (Figure 5). In both heated treatments in ‘MP‐29,’ there was no statistically significant difference in H_2_O_2_ levels between hypoxic and normoxic plants (Figure 5). Similarly, in the non‐heated treatments in ‘Flordaguard’, H_2_O_2_ levels were significantly higher in hypoxic than normoxic plants (Figure 5), whereas in ‘MP‐29,’ there was no significant difference in H_2_O_2_ levels between hypoxic and normoxic plants (Figure 5). Overall, in all treatments except the normoxia, non‐heated treatment, H_2_O_2_ levels were consistently higher in ‘Flordaguard’ compared to ‘MP‐29’ (Figure 5).

### 3.5 Leaf antioxidants and osmoprotectants

In the heated and non‐heated treatments for both rootstocks, the SOD activity was higher in hypoxic than normoxic plants (Figure 5). However, in the normoxia, non‐heated treatment, no significant difference in SOD activity was observed between ‘Flordaguard’ and ‘MP‐29’ (Figure 5). In contrast, in the hypoxia, non‐heated treatment, the normoxia, heated treatment, and the hypoxia, heated treatment, SOD activity was significantly higher in ‘MP‐29’ than ‘Flordaguard’ (Figure 5).

In both the heated and non‐heated treatments for both rootstocks, the POD activity was significantly higher in hypoxic than normoxic plants (Figure 5). In the non‐heated, normoxia treatment and the hypoxia treatment, there was no significant difference in POD activity between ‘Flordaguard’ and ‘MP‐29’ (Figure 5). In the hypoxia and normoxia, non‐heated treatments, POD activity was significantly higher in ‘MP‐29’ compared to ‘Flordaguard’ (Figure 5).

In both the heated and non‐heated treatments for both rootstocks, CAT activity was significantly higher in hypoxic plants compared to normoxic plants (Figure 5). However, in the non‐heated, normoxia treatment, there was no significant difference in CAT activity between ‘Flordaguard’ and ‘MP‐29’ (Figure 5). Conversely, in the hypoxia, non‐heated treatment, the normoxia, heated treatment, and the hypoxia, heated treatment, CAT activity was significantly higher in ‘MP‐29’ compared to ‘Flordaguard’ (Figure 5).

In both the heated and non‐heated treatments for both rootstocks, the APX activity was significantly higher in hypoxic than normoxic plants (Figure 5). Also, in the non‐heated, normoxia treatment, there was no significant difference in APX activity between ‘Flordaguard’ and ‘MP‐29’ (Figure 5). In contrast, in the hypoxia, non‐heated treatment, the normoxia, heated treatment and the hypoxia, heated treatment, APX activity was significantly higher in ‘MP‐29’ compared to ‘Flordaguard’ (Figure 5).

In both the heated and non‐heated treatments for both rootstocks, the GPX activity was significantly higher in hypoxic than normoxic plants (Figure 5). In all treatments, there was no significant difference in GPX activity between ‘Flordaguard’ and ‘MP‐29’ (Figure 5).

In both the heated and non‐heated treatments for both rootstocks, proline levels were significantly higher in hypoxic plants than normoxic plants (Figure 5). In the normoxia, non‐heated treatment, there was no significant difference in proline levels between ‘Flordaguard’ and ‘MP‐29’ (Figure 5). In contrast, in the hypoxia, non‐heated treatment, the normoxia, heated treatment, and the hypoxia, heated treatment, proline levels were significantly higher in ‘MP‐29’ compared to ‘Flordaguard’ (Figure 5).

In both the heated and non‐heated treatments for both rootstocks, the GB levels were significantly higher in hypoxic than normoxic plants (Figure 5). Moreover, in all treatments, GB levels were consistently higher in ‘MP‐29’ compared to ‘Flordaguard’ (Figure 5).

In both the heated and non‐heated treatments for both rootstocks, LPO levels were significantly higher in hypoxic than normoxic plants (Figure 5). In all treatments, ‘Flordaguard’ had significantly higher LPO levels than ‘MP‐29’ (Figure 5).

## 4 DISCUSSION

The results of this study revealed significant differences in net photosynthesis, stomatal conductance, and transpiration between *Prunus* rootstocks in normoxic versus hypoxic root zone conditions. Also, there were treatment differences in physiological and biochemical response to root zone temperature. This suggests a relationship between rootstock physiology and environmental stress, posing questions about the underlying mechanisms driving these variations. Our observation of increased net photosynthesis of ‘MP‐29’ under hypoxia, high temperature, or their combination hints at a potential adaptive advantage. This prompts further investigation into the genetic or physiological factors that confer greater stress resilience in ‘MP‐29’, shedding light on strategies for enhancing productivity in challenging root environments. McGee et al. (2021) reported higher net CO_2_ assimilation in ‘MP‐29’ compared to ‘Flordaguard’ when plants were exposed to 7 days root zone flooding. While this study did not directly assess root zone oxygen levels, the observed physiological responses suggest a probable hypoxic environment, given that, oxygen diffuses about 10,000 times slower in water than in air (Armstrong, 1980; Ferrell & Himmelblau, 1967; Xing et al., 2014). Further research, assessing root zone oxygen levels and establishing thresholds for identifying hypoxic conditions, would strengthen our understanding of root zone dynamics and their significance in bolstering plant resilience. The ability of ‘MP‐29’ to assimilate CO_2_ when exposed to water stress is crucial for maintaining carbohydrate production and providing energy necessary for the functioning of stress response mechanisms and cellular functioning within the plant. When photosynthetic activity is impaired, plants rely on carbohydrate stores to continue their normal functioning (Groszmann et al., 2016; Haupt‐Herting & Fock, 2002; Weis & Berry, 1988; Wong et al., 1985). While maintenance of carbohydrate production may support the plant through a stress event, weakened physiological systems may not fully recover or recover at all following the stress event, leading to premature tree death (Reighard et al., 2001).

Morphologically, ‘Flordaguard’ exhibited an immediate response to root zone stress, showing more severe wilting in plants exposed to the combination of hypoxia and heat. Under normoxic, heated conditions ‘Flordaguard’ wilted more visibly than ‘MP‐29’. Wilting and subsequent defoliation were observed in ‘Flordaguard’ plants after 24 h. After the first 24 h, ‘MP‐29’ began to wilt, but defoliated at a slower rate compared to ‘Flordaguard’ throughout the trial period. During the recovery period, stem and shoot turgidity was greater in ‘MP‐29’ compared to ‘Flordaguard’. Overall, ‘Flordaguard’ struggled to recover, with complete defoliation occurring in several plants.

At the end of the study, the root systems of each plant were collected and examined. Root systems of both rootstocks exposed to the combination of hypoxia and heat were much shorter, dark in color, and showed less overall branching. Although of that group, ‘MP‐29’ had a greater amount of new growth compared to ‘Flordaguard’ which had virtually no new growth. Root systems of both rootstocks exposed to ambient temperatures and hypoxia developed poorly structurally, having very long roots with few lateral extensions. For root systems exposed to normoxia and heat, ‘MP‐29’ had greater lateral development compared to ‘Flordaguard’, although both rootstocks exhibited stunted vertical growth.

Exploring the carbohydrate partitioning mechanism within ‘MP‐29’ could help to develop *Prunus* rootstocks tolerant of environmental stressors that typically are detrimental to leaf gas exchange. The lack of significant differences in intercellular CO_2_ concentrations among treatments may be a result of the juvenile age of the plants used in this study. Older plants, as suggested by Adams (2023) and Sgamma (2017), might possess richer energy stores, supplementing greater carbohydrate requirements during stress events. Juvenile plants, as demonstrated by Smith et al. (2012), exhibit resilience to environmental stress, with their physiological responses more strongly influenced by developmental stage. The establishment of functional stomata and other essential anatomical features for efficient gas exchange takes precedence. Furthermore, as highlighted by Brown and Jones (2017), young plants often prioritize resource allocation towards structural growth and establishment, fostering resilience to environmental stressors. This strategic allocation may mitigate stress impacts on intercellular CO_2_ concentration, as resources are directed to maintain vital functions. Bacelar et al. (2012) study further supports these findings, noting that juvenile plants tend to adopt a conservative water‐use strategy under stress conditions, contributing to the stability of intercellular CO_2_ concentration through regulated stomatal conductance and minimized water loss. Further research is needed to explore the interactions between developmental stages and environmental stressors for a more comprehensive understanding of stress response in developing plants. Understanding the connection between stress response and developmental stage in *Prunus* rootstocks will provide valuable information to amend planting schedules, so that seasonal factors (high temperatures, heavy precipitation, severe weather events, etc.) do not hinder establishment.

Stomatal conductance was found to be affected by environmental stress factors and influenced by both internal and external plant temperature. In the present study, stomatal conductance significantly decreased after the first 24 h of exposure to root zone stress, including hypoxia, heat, and the combination of the two. There were no differences in stomatal conductance between ‘Flordaguard’ and ‘MP‐29’ across all treatments. This observation contradicted the results of McGee et al. (2021), who reported higher stomatal conductance in ‘MP‐29’ after prolonged flooding. This observed reduction in stomatal conductance, coupled with the lack of stress‐induced changes in internal CO_2_ concentration, contrasts with previous studies linking stomatal conductance with increased cellular heat (Urban et al., 2017). Measurements were made between 1000 and 1200 HR each day, when the peak daily temperature typically occurs, possibly exacerbating stomatal sensitivity to Florida's subtropical climate, as noted by McGee et al. (2021). McGee et al. (2021), found that ‘MP‐29’ had higher stomatal conductance than ‘Flordaguard’ at ambient root zone temperatures, but further investigation is required to determine how high root zone temperatures impact stomatal conductance. Net photosynthesis may indirectly drive stomatal conductance as the production of sugars creates a hypertonic osmotic environment within guard cells, promoting water uptake and turgor pressure opening stomata to facilitate gas exchange and water vapor loss through transpiration (Gago et al., 2016). Under hypoxic conditions and ambient root temperatures, ‘MP‐29’ exhibited higher transpiration rates than in ‘Flordaguard,’ indicating a greater tolerance of ‘MP‐29’ to hypoxia‐induced stress. There were no other significant differences between rootstocks in transpiration found in any of the other treatment groups. While relocating peach orchards further inland may offer protection against coastal storm intensity, reduced wind activity could hinder gas exchange, impacting overall orchard health. Wind flow through the orchard is critical to circulating humidity encompassing the canopy that can block gas exchange (Zhu et al., 2022). The gas exchange mechanisms of the juvenile plants used in the present study may have been unable to adapt to the stagnant hot, humid greenhouse atmosphere conditions (Fanourakis et al., 2020). Additionally, the inefficient stomatal conductance of these juvenile plants may have contributed to lower intercellular CO_2_ concentrations, effectively hindering assimilation rates. Further research into the separate and concurrent impacts of developmental age, plant location, and environmental weather conditions may elucidate how these factors can support optimized leaf gas exchange under severe weather conditions.

No significant differences were observed in water use efficiency among treatments nor rootstocks, which can be further explored to identify the genetic traits or specific mechanisms responsible for this equilibrium following the initial drop to improve water regulation under high temperature and subsequent water stress during Florida summer months (Adhikari et al., 2022; Shivran et al., 2022). It is important to note that despite the lack of significant differences between rootstocks and among treatments, water use efficiency was very low, and reflected the plant's inability to manage antagonistic stresses at a juvenile developmental stage.

In the present study, ‘MP‐29’ had higher Fv/Fm and leaf chlorophyll index when exposed to hypoxia. However, when subjected to a combination of hypoxia and high root zone temperature, both variables were higher in “Flordaguard than ‘MP‐29’”. Under normoxic conditions and high temperature conditions in the root zone, there was no significant difference in Fv/Fm between ‘MP‐29’ and ‘Flordaguard,’ but the leaf chlorophyll index was higher for ‘Flordaguard’. The differences in chilling requirement between’MP‐29‐ and ‘Flordaguard’‐ approximately 750 and 300 h, respectively‐suggest potential variations in genetics related to root zone temperature sensitivity between the two rootstocks (Beckman et al., 2012; Sherman et al., 1991). This hypothesis gains further support from the higher Fv/Fm observed in ‘MP‐29′ compared to ‘Flordaguard’ under control conditions, where root zone temperatures remained around 31.8°C, nearly 22º cooler than the maximum temperatures recorded in the hypoxia and heat treatments. Photosystem II plays a crucial role in energy capture, electron transport, and oxygen production during photosynthesis (Barber, 2014). Under temperature stress, the functioning of photosystem II can be strained, leading to reduced carbon assimilation and growth inhibition due to damage such as protein denaturation and disruption of pigment protein complexes (Agrawal & Jajoo, 2021; Choi et al., 2016; Vosnjak et al., 2022). Ferguson et al. (2020) reported high temperature tolerance in juvenile rice plants was a function of non‐photorespiratory release of CO_2_ rather than the photosynthetic capacity, emphasizing the importance of carbon assimilation in the genetic development of heat tolerant rootstocks. The higher Fv/Fm observed in ‘MP‐29′ under control conditions compared to ‘Flordaguard’, along with higher leaf chlorophyll index under hypoxic conditions, suggests that ‘MP‐29′ may possess a higher efficiency of PSII than ‘Flordaguard’. However, the lack of significant differences in Fv/Fm between ‘MP‐29′ and ‘Flordaguard’ in the presence of high root zone temperatures indicates that the imposed stresses may have surpassed both rootstocks' thresholds to tolerate them. Haque et al. (2014) demonstrated that pre‐acclimation to high temperatures (15 and 25ºC) can enhance plant resilience to heat stress (40ºC), suggesting a potential benefit for ‘MP‐29′ given its observed efficiency of PS II. Priming ‘MP‐29′ rootstocks by exposing them to root zone hypoxia and high temperatures during the juvenile stage, prior to field establishment could enhance tree longevity and tolerance to the combination of root zone hypoxia and high temperature experienced in severe weather events during summer months. Studies concerning heat and water stress priming of root systems have reported greater resiliency of the photosynthetic system, enhanced water use efficiency, and upregulation of heat shock gene expression in young plants (He et al., 2022; Ru et al., 2022; Vincent et al., 2020). These findings align with those of McGee et al. (2021), who also reported consistently higher leaf chlorophyll index in ‘MP‐29′ plants compared to ‘Flordaguard’ under flooded conditions, indicating a potential advantage of ‘MP‐29′ in maintaining chlorophyll production, a vital component of the photosynthetic process under oxygen depleted conditions in the root zone.

In the present study, we observed significantly higher SOD, CAT, and APX activities in ‘MP‐29’ than in ‘Flordaguard’ when exposed to root zone hypoxia, indicating an enhanced antioxidant defense response of ‘MP‐29’ (Gill & Tuteja, 2010). These antioxidant enzymes play crucial roles in scavenging ROS and protecting plant cells from oxidative damage induced by stress conditions (Mittler et al., 2004). Conversely, there was no significant difference in POD and GPX activities between the two rootstocks under hypoxic conditions, suggesting that these enzymes may not be as directly involved in the antioxidant response to hypoxia (Mittler et al., 2004). Higher levels of SOD, POD, CAT, and APX in ‘MP‐29’, under high root zone temperature, indicates greater root zone thermotolerance compared to ‘Flordaguard’. The simultaneous exposure to hypoxia and high temperature resulted in higher SOD, POD, CAT, and APX levels in ‘MP‐29’ compared to ‘Flordaguard’, highlighting ‘MP‐29's capacity for integrated stress management (Mittler et al., 2004). Despite consistently higher GPX levels in ‘MP‐29′ in all treatments, there was no significant difference in GPX compared to ‘Flordaguard’, encourages further exploration into the function of GPX in the differential stress response between the two rootstocks. These findings are consistent with McGee et al. (2021), who reported that hypoxia induced enzymatic antioxidant activity in both ‘Flordaguard’ and ‘MP‐29′ under flooded conditions. Similarly, our study revealed that ‘MP‐29′ had higher enzymatic antioxidant activity than ‘Flordaguard’ when exposed to hypoxia in combination with high temperature in the root zone, indicating a potentially more efficient response to oxidative stress in ‘MP‐29’.

In addition to higher antioxidant enzyme activities, ‘Flordaguard’ and ‘MP‐29’ displayed the highest osmolyte content when exposed to hypoxia and high temperature in the root zone. McGee et al. (2021) also observed higher proline and glycine betaine contents in ‘MP‐29’ compared to ‘Flordaguard’ under flooded root zone conditions, which were assumed to be hypoxic. The accumulation of osmolytes, such as proline and glycine betaine, may play a vital role in protecting photosystem II, enhancing the stability of the photosynthetic process, and contributing to osmotic adjustment and ROS scavenging throughout the stress period (Hare & Cress, 1997; Oukarroum et al., 2012). Furthermore, in our study, ‘Flordaguard’ had significantly higher LPO levels compared to ‘MP‐29’ across all treatments, indicative of increased oxidative damage in ‘Flordaguard’ (Jogawut et al., 2019; Lamiae et al., 2021). This observation aligns with our expectation of lower LPO in ‘MP‐29’ due to its higher antioxidant activity. Similarly, our findings are in line with McGee et al. (2021), who reported higher lipid peroxidation in ‘Flordaguard’ compared to ‘MP‐29’ during flooding. Moreover, ROS contents (O_2_ and H_2_O_2_) were higher in ‘Flordaguard’ across all treatments compared to ‘MP‐29’. The combination of lower lipid peroxidation and higher antioxidant activity in ‘MP‐29’ suggests that it may better tolerate oxidative stress induced by hypoxia, heat, or their combination in the root zone compared to ‘Flordaguard’ (Akbari et al., 2022; Lamiae et al., 2021).

In the present study, leaf nutrient analysis revealed minimal divergence in leaf nutrient element concentrations between ‘Flordaguard’ and ‘MP‐29’, with notable exceptions for N, P, and K. ‘Flordaguard’ consistently exhibited higher N concentrations across all treatments compared to ‘MP‐29’. Additionally, significant differences in P and K were found in the normoxic, non‐heated and heated treatments, where ‘Flordaguard’ displayed higher levels of these nutrients relative to ‘MP‐29’. N, P, and K are integral components in the maintenance of the photosynthetic system, regulation of stomatal function, and osmotic potential in plants (Hou et al., 2019; Lu et al., 2019; Zangani et al., 2021). Nitrogen is a crucial constituent of chlorophyll molecules, essential for light absorption and carbon assimilation during photosynthesis (Hou et al., 2019). Phosphorous plays a vital role in ATP synthesis, serving as an energy currency for various metabolic processes, including photosynthetic electron transport and carbon fixation (Zangani et al., 2021). K regulates stomatal opening and closure, thereby influencing gas exchange and water regulations in plants (Lu et al., 2019).

When exposed to hypoxia, ‘MP‐29’ had higher Mg, Ca, and S compared to ‘Flordaguard’. The higher Ca levels in ‘MP‐29’ may play a critical role in cellular membrane fortification, protecting cell walls from cytoplasm acidification during anaerobic fermentation (Thor, 2019). Additionally, Mg and S are essential for chlorophyll synthesis, which is vital for light absorption to maintain photosynthetic efficiency and cellular respiration in response to stress (Ishfaq et al., 2022; Narayan et al., 2023). For plants exposed to hypoxia and high temperature, ‘MP‐29’ had significantly higher B ppm compared to ‘Flordaguard’. Boron facilitates sugar transport within plant vasculature and optimizes carbohydrate allocation, especially during periods of external stress (Pommerrenig et al., 2019; Rajesh et al., 2021). This heightened B accumulation in ‘MP‐29’ may contribute to improved carbohydrate mobilization and allocation, essential for sustaining metabolic processes and stress resilience in fruit trees. When exposed to root zone conditions such as hypoxia or high temperature stress, fruit trees significantly shift resource prioritization favoring essential functions, resulting in reduced carbohydrate mobilization to roots and a slowdown in root growth (Vartapetian, 2006; Loreti & Perata, 2020; Salvatierra et al., 2020; Habibi et al., 2023). This shift affects various root tissues, with fine roots, responsible for nutrient water and uptake, receiving less energy (Pregitzer, 2008). Stress also impacts root respiration rates, with some stressed roots exhibiting increased respiration to cope, yet excessive respiration can deplete stored carbohydrates, affecting overall plant health (Huang et al., 2012; Jethva et al., 2022). Roots may adapt by adjusting metabolic pathways and carbohydrate utilization (Kang et al., 2019; Karlova et al., 2021; Raza, 2020). The interconnected nature of carbohydrate allocation between roots and shoots creates a feedback loop, whereby stressed roots influence shoot growth and vice versa, shaping overall plant development (Xu et al., 2015; Kang et al., 2019). For other micronutrients (Zn, Mn, Fe, and Cu), no significant differences were found between ‘Flordaguard’ and ‘MP‐29’ across all treatments. However, variations in these elements within each rootstock and between treatments suggest that the genetic background and the impact of stress factors prompted differences in nutrient uptake.

Further trials are needed to explore the behavior of these two rootstocks in the production environment with the addition of rootzone stress. Limitations of such a controlled hydroponic design within the greenhouse environment are the absence of mechanical damage, pathogen pressure, and hazardous chemical release typically experienced in severe weather events. The volatility of a severe weather event may depend on various factors including proximity to coastal waters, topography of the orchard, and site preparation. Another limitation of the hydroponic deep‐water bucket design is the lack of substrate and growth area afforded by in‐ground cultivation. The design did not account for potential soil erosion due to heavy precipitation and high wind speeds that may leave root systems vulnerable to surface‐level conditions prior to flood water accumulation.

Additionally, the added factor of scion grafting must be explored to fully understand the tolerance of these rootstocks to heat and hypoxia in the root zone. Physiological measurements were collected from the scion of these rootstocks, which may not reflect the performance seen of a differing grafted scion in the production environment. Current literature suggests that scion‐rootstock combinations can have significant impacts on carbohydrate partitioning, canopy development, and physiological performance (Bir & Ranney, 2016; Montesinos et al., 2024; Zhu et al., 2024). Finding the appropriate scion‐rootstock combination is crucial to achieve maximum tolerance to root zone stressors experienced during a severe weather event.

## 5 CONCLUSIONS

Under combined hypoxic and heated root zone conditions, ‘MP‐29’ outperformed ‘Flordaguard’ biochemically. The higher antioxidant activity, osmolyte contents, lower lipid peroxidation, and lower ROS contents in ‘MP‐29’ compared to ‘Flordaguard’ indicate its greater tolerance to oxidative stress resulting from oxygen depletion and subsequent water stress in the root zone. Additionally, under high temperatures in the root zone, ‘MP‐29’ had higher leaf N, P, and K concentrations than ‘Flordaguard’. These are essential nutrients for physiological processes and plant growth. The higher concentrations of Ca and B in ‘MP‐29’ when exposed to hypoxia and high temperatures may have contributed to its greater turgidity. The more efficient nutrient absorption in ‘MP‐29’ suggests greater root tolerance to hypoxia and high temperatures. Additionally, ‘MP‐29’ exhibited superior physiological performance than ‘Flordaguard’ under either hypoxic or heated root zone conditions. However, when these stressors were combined, ‘Flordaguard’ showed better physiological performance than ‘MP‐29’.

The findings of this study have significant implications for the selection of *Prunus* rootstocks in Florida and other regions facing similar environmental challenges. ‘MP‐29’ emerges as a promising rootstock, demonstrating greater biochemical performance under hypoxia and heated root zone conditions. Enhanced antioxidant activity, osmolyte content, and nutrient absorption capabilities make it a valuable candidate for crops in a challenging environment. Nevertheless, it is important to acknowledge that ‘Flordaguard’ performs better physiologically than ‘MP‐29’ under the combined stresses of root zone hypoxia and high temperature, emphasizing the need for a thorough approach to rootstock selection in diverse environmental conditions. This study alone does not substantiate the superiority of ‘MP‐29’ compared to ‘Flordaguard’ in terms of tolerance to root zone heat and hypoxia. Careful consideration of these findings can contribute to the optimization of crop yields and promote sustainable food production in Florida and in other regions susceptible to flooding during periods of high temperatures.

## CONFLICT OF INTEREST STATEMENT

The authors declare that they have no conflict of interest.

## Data Availability

The data that support the findings of this study are available on request from the corresponding author. The data are not publicly available due to privacy or ethical restrictions.
